# Synthesis of Silver Nanocomposites for Stereolithography: In Situ Formation of Nanoparticles

**DOI:** 10.3390/polym14061168

**Published:** 2022-03-15

**Authors:** Luisa M. Valencia, Miriam Herrera, María de la Mata, Alberto S. de León, Francisco J. Delgado, Sergio I. Molina

**Affiliations:** Departamento de Ciencia de los Materiales e Ingeniería Metalúrgica y Química Inorgánica, IMEYMAT, Facultad de Ciencias, Universidad de Cádiz, Campus Río San Pedro, s/n, Puerto Real, 11510 Cádiz, Spain; miriam.herrera@uca.es (M.H.); maria.delamata@uca.es (M.d.l.M.); alberto.sanzdeleon@uca.es (A.S.d.L.); fjavier.delgado@uca.es (F.J.D.); sergio.molina@uca.es (S.I.M.)

**Keywords:** additive manufacturing, stereolithography, polymer-based nanocomposites, acrylic resin, silver nanoparticles, in situ generation

## Abstract

Additive Manufacturing (AM) offers remarkable advantages in relation to traditional methods used to obtain solid structures, such as the capability to obtain customized complex geometries adapted to individual requirements. The design of novel nanocomposites suitable for AM is an excellent strategy to widen the application field of these techniques. In this work, we report on the fabrication of metal/polymer nanocomposites with enhanced optical/electrical behaviour for stereolithography (SLA). In particular, we analyse the in situ generation of Ag nanoparticles (NPs) from Ag precursors (AgNO_3_ and AgClO_4_) within acrylic resins via SLA. Transmission electron microscopy (TEM) analysis confirmed the formation of Ag NPs smaller than 5 nm in all nanocomposites, providing optical activity to the materials. A high density of Ag NPs with a good distribution through the material for the larger concentration of AgClO_4_ precursor tested was observed, in contrast to the isolated agglomerations found when the precursor amount was reduced to 0.1%. A significant reduction in the electrical resistivity up to four orders of magnitude was found for this material compared to the unfilled resin. However, consumption of part of the photoinitiator in the formation process of the Ag NPs contributed to a reduction in the polymerization degree of the resin and, consequently, degraded the mechanical properties of the nanocomposites. Experiments with longer curing times showed that, for the higher AgClO_4_ concentrations tested, post-curing times of 300 min allowed an 80% degree of polymerization to be achieved. These conditions turned these materials into promising candidates to obtain solid structures with multifunctional properties.

## 1. Introduction

Additive Manufacturing (AM) is gaining interest and is experiencing a significant evolution in industry due to a number of advantages including customization and complex geometries [1,2,3,4,5,6,7,8]. AM techniques consist of the fabrication of 3D objects of any shape by the sequential deposition of material layers [9]. Initially, this technology was used to produce prototypes before large-scale production [10,11], but nowadays it is also suitable for the fabrication of final pieces intended for a range of broad applications, covering sectors such as optics [12], electronics [13], and medicine [14], among others.

Within AM techniques, those using photocurable resins are expanding their application field. Stereolithography (SLA) and Digital Light Processing (DLP) are particularly interesting due to the high resolution and smooth surfaces obtained in the final printed objects compared to other AM techniques, such as Fused Filament Fabrication (FFF) [1,15]. SLA was developed in the 1980s by 3D Systems (Valencia, CA, USA) and it is a tank-based technique where a photocurable liquid resin is solidified by local radiation (ultraviolet laser, UV) with a certain wavelength. This technique allows a high level of accuracy to be achieved through the use of thinner deposition layers which are able, for instance, to reduce the porosity [16,17].

In order to expand the application field of SLA, research has been intensified in the last few years to obtain new composite materials with tailored properties that can be processed by this technology [2,4,5,18], for example by adding metal or ceramic phases to improve their mechanical and functional properties [19,20,21]. Within this context, there are reported works that focus on the incorporation of different types of nanoparticles (NPs) in commercially available acrylate resins to modify the electrical [22], optical [23], mechanical [24], and thermal [25] behaviour, among others. Metallic NPs offer appealing physicochemical properties, i.e., optical, and electronic properties, due to their high surface area to volume ratio. In particular, the optical properties of Ag NPs have been historically exploited in decorative pigments for jewellery/handicrafts, staining glass, or ceramics [26,27,28,29]. Over the last decades, Ag NPs have found use in many areas, such as catalysis [30,31], optical and electro-optical devices [23,32], surface-enhanced Raman spectroscopy (SERS) [33,34], and antimicrobial agents [14,35]. Ag NPs are promising nanofillers to obtain composites with electrical and optical properties to extend the applicability of SLA including the electronic or biomedical fields [13,14,36,37,38,39].

Processing hybrid (metal/polymer) compound materials by SLA involves taking into account further considerations. The addition of NPs to the resin could modify the solution viscosity and the light penetration depth, affecting the photopolymerization of the resin that occurs during the printing process and degrading the quality of final pieces. The sedimentation of NPs during the process could also occur which is detrimental for the homogeneity of the macroscopic properties of solid printed objects. Some of these issues could be overcome by generating the Ag NPs in situ within the polymer during the printing process, where the photoreduction of a metal precursor is coupled to the photopolymerization of the acrylic matrix. In situ approaches have been considered, for example, to obtain Ag nanocomposite cellulose fabrics with antibacterial activity for hospital bed materials, using simple and environmentally friendly bioreduction/hydrothermal methods for the generation of Ag NPs [40,41]. In situ approaches have also been previously considered in AM techniques. For DLP photopolymers, Fantino et al. [36] reported on the generation of Ag NPs in acrylic resins in a UV curing process applied after a DLP process, resulting in non-homogeneous NPs distributions. These authors also reported on the thermal reduction of Ag NPs within the acrylic resin after the DLP process, which resulted in different electrical conductivities for the different temperatures and times tested [42]. In SLA, customized equipment has been proposed to fabricate structures with silver-patterned surfaces by modifying the laser settings during the 3D printing process, which would be suitable for resistive switching devices [43,44]. Scancialepore et al. [45] proposed the in situ fabrication of 3D printed pieces with Ag NPs using low Ag precursor contents (Ag acetate), obtaining only a slight decrease in the electrical resistivity, and the same order of magnitude of the pristine resin. At the sight of this previous work, optimizing the synthesis parameters and exploring different photocurable resins and Ag precursors from those reported so far in the literature is evidenced to be relevant to reach the desired electrical conductivity in these SLA composites to expand their application field.

The combination of optically and electronically functional materials with the ability of additive manufacturing to create complex 3D geometries allows the creation of devices that are not possible with conventional methods. Examples include multilayer circuit boards, organic light emitting diodes (OLEDs), and biosensors, among others. Understanding the science behind these novel materials is essential to contribute to the development of objects with applications in technologies as relevant to today’s society as the aerospace, automobile, or biomedical industries.

Inspired by the above-mentioned studies, we analysed the effect of using different acrylic resins and Ag precursors to fabricate Ag nanocomposites with tailored optical and electrical properties in a single-step synthesis during the printing process, which broadens the field of study of SLA materials. For that, in the present work, two precursors (AgNO_3_ and AgClO_4_) were compared to achieve the in situ generation of Ag NPs in an acrylic resin by SLA in order to create promising conductive nanocomposites. The resin formulation used contained a photoinitiator responsible for initiating the photopolymerization of the acrylic resin during the printing process. This photoinitiator was also used for the photoreduction of the Ag precursor, which simplified the process and avoided the addition of extra compounds to the resin that could have affected the printing process. Because of this, a competitive reaction for the photoinitiator took place which defined both the optical and electrical properties of the composite (determined by the Ag NPs density) and its mechanical properties (determined by the degree of resin polymerization achieved). The effect of the concentration and nature of the Ag precursors in these properties is evaluated and discussed, together with the effect of increasing the duration of the post-curing treatment to improve the mechanical properties.

## 2. Materials and Methods

### 2.1. Materials

Clear photopolymer standard resin (a mixture of proprietary acrylic monomers and oligomers and phenylbis (2,4,6-trimethyl benzoyl)-phosphine oxide as a photoinitiator) was purchased from XYZprinting, Inc (XYZprinting, New Taipei City, Taiwan). Silver nitrate (AgNO_3_) was purchased from VWR Chemicals, silver perchlorate (AgClO_4_) was purchased from Alfa Aesar, and isopropanol (IPA) was purchased from Scharlau. All products were used as received.

### 2.2. Sample Preparation

Different precursors with 0.1 and 1 wt% AgNO_3_ and 0.1, 1, and 3 wt% AgClO_4_ were prepared to fabricate the composites. An Ultrasonic Cleaner USC500T provided by VWR (VWR International, Radnor, PA, USA) and working at 45 kHz was used for the sonication processes (30 min). Solid specimens were printed by SLA with Nobel 1.0, XYZprinting, Inc. (XYZprinting, New Taipei City, Taiwan), using a 405 nm laser with an output power of 100 mW and a spot size that allowed an XY resolution of 300 µm. Dogbone specimens according to ASTM D638 (type 1BA) for mechanical tests, flat discs of 2 mm thickness and 65 mm diameter for electrical measurements, and monolayers of 0.1 mm thickness and 65 mm diameter for optical testing were printed. Moreover, a complex cubic structure with hollows and curved parts (2 × 2 × 2 cm^3^) was also made. All samples were printed with a layer height of 100 µm. Once printed, the samples were washed in IPA for several minutes. Post-processing of the samples was performed inside a UV chamber with a light source of 405 nm and a power of 1.25 mW/cm^2^ (FormCure, Formlabs, Somerville, MA, USA) heated at 60 °C for 60, 180, and 300 min. Small pieces of the dogbone specimens were used for the DSC measurements.

### 2.3. Characterization

Electrical resistivity was measured following ASTM D257 using a Keithley 6517B electrometer (Keithley, Cleveland, OH, USA) with a voltage of 500 V. At least three measurements were performed for each composite. The results were averaged with standard deviations presented as error bars.

UV-Vis spectra were measured using a Varian Cary 50 Conc spectrophotometer. The range between 200 and 800 nm was monitored with a scan rate of 10 nm/s.

Tensile testing of at least five specimens of each nanocomposite was performed in a Universal Testing Machine (Shimadzu, Japan) at a constant speed of 1 mm/min, according to ASTM D638. The results were averaged and standard deviations were presented as error bars.

The curing enthalpy of the different samples was determined by Differential Scanning Calorimetry (DSC) with a Q20 (TA Instruments, New Castle, DE, USA). DSC curves were obtained by performing a temperature sweep from room temperature (25 °C) to 320 °C at 10 °C/min under a nitrogen atmosphere. A subsequent cooling and heating sweep at 10 °C/min was performed to confirm the complete polymerization of the resin in the first sweep.

Electron-transparent thin films of about 70 nm of the nanocomposites for transmission electron microscopy (TEM) analyses were obtained using a Leica EM UC7 ultramicrotome equipped with a diamond knife.

High angle annular dark field scanning TEM (HAADF-STEM), high resolution TEM (HRTEM), and Energy-dispersive X-ray (EDX) measurements were performed using a Thermo Scientific TALOS F200S (Thermo Fisher Scientific, Waltham, MA, USA) working at 200 kV.

## 3. Results

### 3.1. Fabrication of Nanocomposites

**Figure 1:**
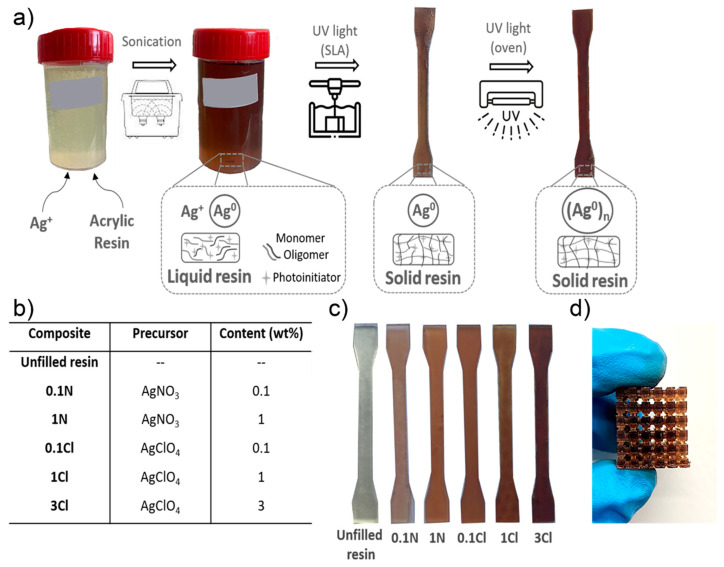
(**a**) Scheme depicting the fabrication of the nanocomposite printed objects using SLA; (**b**) table defining the nanocomposite solutions; (**c**) 3D printed parts of the different nanocomposites: from left to right, unfilled resin, 0.1N, 1N, 0.1Cl, 1Cl and 3Cl; (**d**) 3Cl object printed by SLA.

Three-dimensional objects were fabricated by SLA following the process schematized in Figure 1a. Initially, the formulations were prepared by adding different amounts of Ag NPs precursors (AgNO_3_ or AgClO_4_) to the resin and sonicating the mixture to disperse the precursors and to homogenize the solution. In order to obtain a large number of Ag NPs to increase their effect in the nanocomposite properties, an amount of precursors reaching the solubility limit were considered. The two precursors had a different solubility within the resin. Thus, up to 3 wt% AgClO_4_ approximately dissolved in the resin before a solid precipitate was observed, whereas this amount was reduced to 1 wt% for AgNO_3_. In order to understand the effect of the composition of the Ag NPs precursors in the properties of the nanocomposite, different values up to the solubility limit were considered: 0.1 and 1 wt% for AgNO_3_, and 0.1, 1, and 3 wt% for AgClO_4_. For clarity, from here on the specimens are labelled with a number indicating the precursor concentration and letters pointing to the precursor nature, as shown in the table in Figure 1b. The solutions changed from light yellow to brown after sonication. This colour change suggests that the initial formation of Ag NPs is started by ambient light [46]. However, it was expected that a larger number of NPs would be formed after the printing process, ideally ensuring the complete photo-reduction of the Ag precursor.

Once the solutions were prepared, they were poured into the SLA tank. When the photoinitiator was irradiated with the printer laser, it was activated, triggering the radical polymerization of acrylic monomers (i.e., the curing of the resin) while simultaneously Ag^+^ was reduced into Ag^0^ [47,48,49]. It was observed that all the nanocomposites were successfully printed for all the concentrations tested, as shown in Figure 1c. However, in the case of 3Cl, the polymerization of the resin reached lower degrees of cure, as some objects presented slight irregularities. This could be due to the high amount of Ag precursor used, which may have consumed a large fraction of the photoinitiator involved in the photopolymerization process. Despite this, Figure 1d shows a printed object of sample 3Cl showing the ability to produce complex geometries.

After the fabrication of the nanocomposites, a post-curing process inside an UV chamber at 60 °C for 60 min was applied to increase their polymerization degree. It was observed that the UV post-processing darkened the printed nanocomposites, suggesting that the density of Ag NPs increased. In the following, we focus the analysis on the structural and functional properties of the nanocomposites in the pieces after the UV post-curing process.

### 3.2. Structural and Compositional Characterization

**Figure 2:**
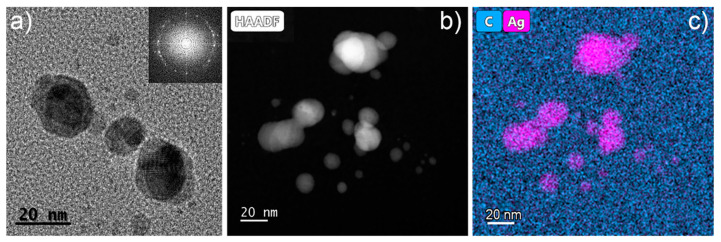
(**a**) HRTEM image of a 3D printed part of the sample 1N. The inset shows the Fast Fourier Transform pattern of the image in (**a**), exhibiting the crystallinity of the nanoparticles; (**b**) HAADF-STEM image of the 3Cl nanocomposite; (**c**) EDX map of the image in (**b**) showing C from the acrylic resin (blue) and Ag from the nanoparticles (pink).

In order to analyse whether Ag NPs were formed within the acrylic resin in the process described above and to understand the effect of the nature and amount of the precursor used, the nanocomposites fabricated were studied by TEM. It must be mentioned that acrylic resins are electron beam-sensitive materials, as are most polymers [50], and because of this care should be taken in their analysis by this technique to avoid artefacts due to deterioration of the material. An exhaustive analysis was carried out to obtain low-dose conditions that allowed the material to be studied, and avoided artefacts due to beam damage and will be published elsewhere. Figure 2a displays a HRTEM image of the 1N sample evidencing the content of the NPs within the acrylic resin. The inset shows the fast Fourier transform (FFT) of the observed NPs, which demonstrates that the NPs were crystalline. Figure 2b shows a HAADF-STEM image of sample 3Cl. Since the HAADF contrast scales with the Z number, the brighter regions observed in Figure 2b are likely Ag NPs. In order to confirm this point, EDX analyses were carried out and are shown in Figure 2c. In EDX, Ag and C mapped signals are displayed in pink and blue, respectively. These results confirm that Ag NPs were synthetized in situ during the photopolymerization of the resin by SLA. Ag NPs were observed in the nanocomposites fabricated using both the AgNO_3_ and the AgClO_4_ precursors.

**Figure 3:**
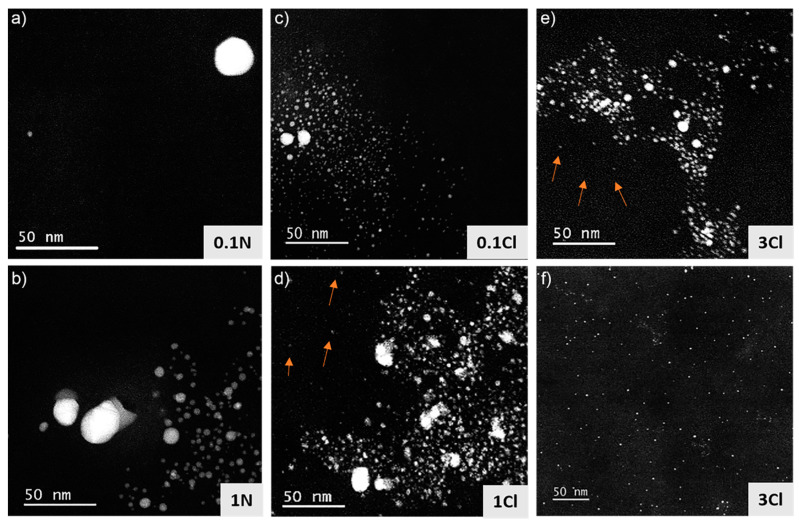
HAADF-STEM images of 3D printed structures containing acrylic resin and different concentrations of silver precursor. (**a**) 0.1N; (**b**) 1N; (**c**) 0.1Cl; (**d**) 1Cl; (**e**) 3Cl; (**f**) 3Cl (different area).

With the aim to understand the effect of the nature and concentration of the precursor on the characteristics of the Ag NPs formed, TEM analysis was carried out in all the nanocomposites fabricated, and the results are shown in Figure 3. Figure 3a shows a HAADF-STEM image of the 0.1N sample. In general, a very low density of NPs was found in this composite. Figure 3b, corresponding to the 1N specimen, shows a larger amount of Ag NPs, which was expected due to the increased precursor concentration used (10 times larger). Interestingly, the size of the NPs was quite small, with approximately 80% of the NPs with a size smaller than 5 nm. However, the distribution of the NPs was not homogeneous; there were coexisting neighbouring areas with a high and low density of NPs. This was likely due to a heterogeneous distribution of the precursor in some areas of the polymer due the high viscosity of the resin that complicated the dispersion of the precursor by sonication, giving rise to the aggregation of Ag NPs. Figure 3c,d show HAADF-STEM images of the 0.1Cl and 1Cl composites, respectively. As can be observed, more NPs were formed when AgClO_4_ was used compared to AgNO_3_, and again there was a higher density when the precursor concentration was increased. The formation of a higher amount of Ag NPs with AgClO_4_ than with AgNO_3_ could be related to the better solubility of this precursor in the acrylic resin mentioned earlier, facilitating the interaction with the photoinitiator present in the acrylic resin. On the other hand, for both precursors, noticeable agglomerations of NPs were found in the composites. However, it is worth highlighting that for 1Cl, besides the agglomeration of NPs, isolated NPs were observed and are marked with arrows in Figure 3d. A homogeneous dispersion of isolated NPs is highly desirable to enhance the macroscopic electrical/optical properties of the material. Figure 3e,f show HAADF-STEM images of the 3Cl nanocomposite. Figure 3e shows an agglomeration of Ag NPs, analogous to those observed in the other nanocomposites studied. However, in this sample, in addition to these agglomerates, homogeneously distributed NPs with small diameters (<5 nm) were found through the material, as is clearly shown in Figure 3f. This distribution is very promising for the enhancement of the electrical/optical properties of the nanocomposites, suggesting uniform properties. Sciancalepore et al. [45] also obtained a good distribution of Ag NPs within an acrylate resin (of different composition than the one used in this work) by SLA using silver acetate as a Ag precursor. However, the larger size of the NPs obtained (10–25 nm) in that work entailed a lower density of NPs for the same amount of Ag precursor, which hindered the achievement of the percolation threshold for electrical conductivity. Additionally, the authors found that silver acetate precipitated at concentrations above 1 wt%, analogously to the results obtained here for AgNO_3_. Non-uniform distributions of larger Ag NPs have also been observed for DLP in situ Ag NPs generation [36].

### 3.3. Analysis of the Electrical and Optical Properties

**Figure 4:**
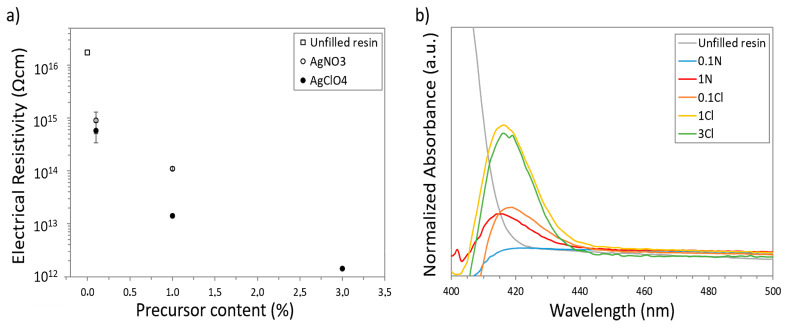
(**a**) Electrical resistivity and (**b**) UV-Vis spectra of the different Ag nanocomposites after the post-curing process.

Electrical measurements were performed on the nanocomposites in order to evaluate the possible variations produced in the volumetric resistivity due to the formation of the Ag NPs observed by TEM. For these analysis, disks of the composites containing the Ag precursors with the concentrations considered were printed. Figure 4a shows the values of the electrical resistivity measured for each nanocomposite after the post-curing process. The pure acrylic resin (reference specimen, without Ag precursors, from now on referred to as *unfilled resin*) presented an electrical resistivity in the range of 10^16^ Ωcm, which means it had a strong insulating behaviour. As can be observed, when the amount of Ag precursors increased, the electrical resistivity decreased as expected due to the high conductivity of metallic NPs. In the case of nanocomposites with AgNO_3_, an improvement in the electrical resistivity of more than one order of magnitude when adding 0.1 wt% (0.1N specimen) and up to two orders of magnitude when adding 1 wt% (1N specimen) was noticed. For AgClO_4_ nanocomposites, a major enhancement in the electrical properties was observed. Thus, a decrease in the electrical resistivity of more than one order of magnitude for 0.1Cl nanocomposites was found, and a decrease of three orders of magnitude for 1Cl nanocomposites. Remarkably, the electrical resistivity reduced four orders of magnitude to 10^12^ Ωcm when 3 wt% AgClO_4_ was added to the acrylic resin (3Cl specimen). These variations are clearly related to the amount and distribution of Ag NPs observed by TEM in each nanocomposite (determined by the concentration and solubility of each precursor), where composites 1Cl and 3Cl showed a better distribution of Ag NPs through the material and, consequently, a stronger reduction in electrical resistivity was found. However, the electrical resistivity values measured still correspond to insulator materials rather than to semiconductor/conductor materials. This is partially due to the extremely high electrical resistivity of the unfilled resin used (10^16^ Ωcm, as mentioned above). Fantino et al. [36] recently reported on the fabrication of conductive nanocomposites by introducing Ag NPs in polyethylene glycol diacrylate (PEGDA) as a polymer matrix, which has an initial electrical resistivity of 10^8^ Ωcm. Here, by decreasing the electrical resistivity only three orders of magnitude, it fell into the range of dissipative/resistive materials. Thus, our results are very promising for resins with lower initial values of electrical resistivity. For the specific acrylic resin used in this work, a larger amount of Ag NPs would be needed to achieve the desired electrical conductivity. As solubility has been shown to be a limiting factor to increase the amount of Ag precursor used, experiments are in progress to evaluate the effect of using intermediate solvents to increase the amount of precursor that can be dissolved in the resin, as this could increase the density of Ag NPs.

On the other hand, UV-Vis spectrophotometry measurements were carried out to analyse the optical characteristics resulting from the formation of the Ag NPs observed by TEM in the acrylic resin. Ag NPs have a characteristic absorption band in the range 400–450 nm corresponding to the oscillation of the conduction electrons that takes place at the surface of the NPs, normally referred to as Localized Surface Plasmon Resonance (LSPR) [51,52]. Unfortunately, the absorption band of the photoinitiator of the acrylic resin also lies within this spectral region [53]. Therefore, the composite spectra obtained were normalized taking as a reference this band from an unfilled resin used as control. Figure 4b shows the spectra corresponding to the unfilled resin and to the considered nanocomposites. The characteristic peak of the metal NPs around 420 nm appeared in every nanocomposite, meaning that the Ag precursors can tune the optical behaviour of the obtained nanocomposites. In particular, a slight blue-shift of the absorption band for the samples obtained using AgNO_3_ compared to those obtained using AgClO_4_ can be seen. This could be related to small changes in the average size of the NPs in the materials. The size of the NPs has a dramatic effect on the LSPR and, consequently, on the optical properties of the NPs, through different mechanisms [51]. In general, for small NPs (<50 nm), the absorption peak blue-shifts as the NPs size decrease [54,55]. However, for NPs smaller than 10 nm, it has been reported that a strong red-shift occurs when the size decreases [56]. This is in good agreement with our results, where an increase in the average size of the NPs was observed for nanocomposites obtained with AgNO_3_ which could induce the observed slight blue-shift of the absorption band. Additionally, it is worth mentioning that a progressive increase in the normalized absorbance for increasing precursor concentrations was found, which can be related to an increase in the density of the Ag NPs in the material.

### 3.4. Mechanical Characterization

**Figure 5:**
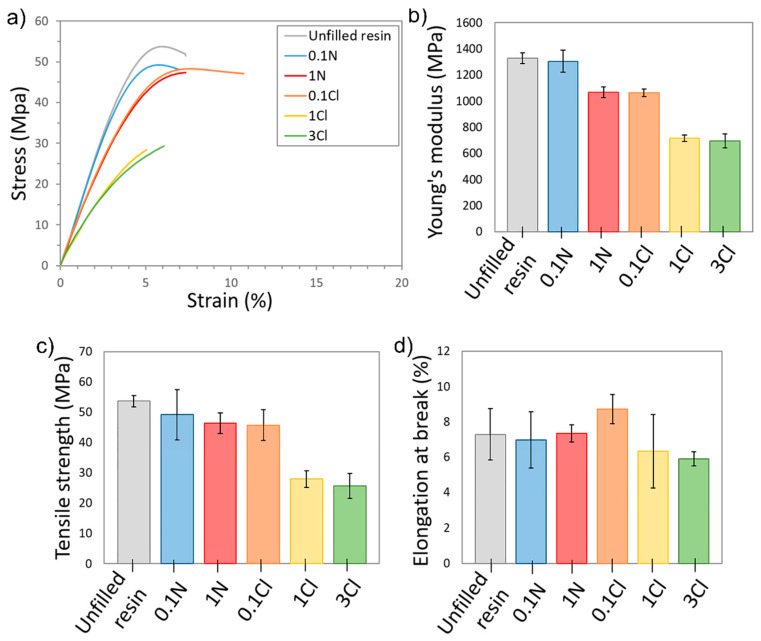
(**a**) Representative stress-strain curves of the samples; (**b**) Young’s modulus; (**c**) tensile strength and (**d**) elongation at break for the Ag nanocomposites studied.

Tensile tests were carried out to analyse the effect of the Ag NPs in the mechanical behaviour of the materials considered in this work. Figure 5a shows representative strain–stress curves corresponding to the different nanocomposites and the unfilled resin. The AgNO_3_ nanocomposites and 0.1Cl curves were quite similar to that of the unfilled resin. However, there was a large difference for the 1Cl and 3Cl composites, where a clear reduction in both Young’s modulus and tensile strength can be observed. This difference is more evident in Figure 5b–d, which show average values of Young’s modulus, tensile strength and elongation at break of the different nanocomposites. While Young’s modulus for 1N or 0.1Cl only decreased around 20–30% with respect to the unfilled resin (whereas tensile strength and elongation at break only present slight variations), for 1Cl and 3Cl nanocomposites there was a drastic reduction of almost 50% both in the Young’s modulus and tensile strength. Analogous results have been previously reported for SLA composites based in acrylic resins with Ag/Cu NPs of larger size [38]. The significant reduction in the mechanical properties observed could be partially due to a poor compatibility between the surface of the Ag NPs and the matrix, which is essential for an additive to be able to act as reinforcement in polymeric composites. This is magnified in the regions of agglomerations of NPs observed by TEM, which could act as potential fracture regions. The irregular distribution of NPs could also be responsible for the large scatter in the mechanical results obtained, as this introduces regions of variable strength in the material. Additionally, the decrease in the mechanical properties observed could be caused by a lower degree of cure of the acrylic resin due to the high amount of Ag precursor present in the nanocomposite, which partially consumes the photoinitiator. The reduction of Ag^+^ to Ag^0^ may affect the degree of cure of the nanocomposites, which can be correlated to their mechanical properties, as we have previously observed in these type of nanocomposites [57].

### 3.5. Analysis of the Degree of Cure of the Nanocomposites

**Figure 6:**
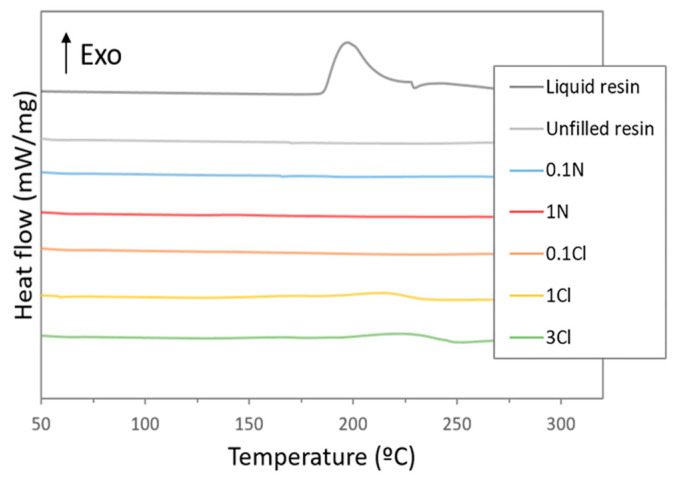
DSC thermograms of the liquid and post-cured resins containing the silver precursors.

To test the influence of the Ag precursor on the photopolymerization of the acrylic resin, thermal analyses of the resins were carried out by DSC. Figure 6 shows the DSC curves for the liquid resin (i.e., totally non-cured) and for the unfilled nanocomposites after the UV post-curing process. The liquid resin presented an exothermic peak at 185–220 °C. This peak corresponds to the *cure enthalpy* (∆H_cure_), since in these conditions it can be assumed that all the monomers of the resin precursor are available for polymerization, and the energy observed corresponds to the thermal polymerization of the acrylic resin. On the other hand, for the 3D-printed and post-cured composites, the values obtained from the DSC sweeps correspond to the *residuary cure enthalpy* (∆H_residuary_) [57]. Hence, the degree of cure can be calculated following Equation (1):

(1) $$\mathrm{Degree}\;\mathrm{of}\;\mathrm{cure}\;(\%)\;=\;\left(1-\frac{\Delta H_{\mathrm{residuary}}}{\Delta H_{\mathrm{cure}}}\right)\times 100$$

As can be observed in Figure 6, the DSC curve of the unfilled resin did not show any noticeable band in the range studied, meaning that the application of a post-processing treatment during 60 min in the UV chamber allows a full degree of cure to be achieved. Similar results were observed for 0.1N, 1N, and 0.1Cl nanocomposites, as the measured DSC thermograms were flat. This seems to indicate that the amount of photoinitiator consumed for the formation of the Ag NPs observed by TEM was not high enough to affect the polymerization of the resin and this is in well-agreement with the mechanical testing results, which showed similar properties than the unfilled resin. However, a small band can be observed in the DSC curves at 200–220 °C for 1Cl and 3Cl nanocomposites, indicating the presence of unreacted monomer in the resin after the post-processing in the UV chamber. In these cases, a degree of cure of approximately 75% was calculated. This could be related to the higher density of Ag NPs observed by TEM in these composites that would require a larger amount of photoinitiator to be synthetized, affecting the photopolymerization of the resin and it is likely to be the reason for the significant decrease in the mechanical properties for these nanocomposites. In general, these results are in agreement with previous research, which show that a decrease in the degree of cure of a photosensitive resin has a direct effect on the mechanical properties of the material [24,57].

**Figure 7:**
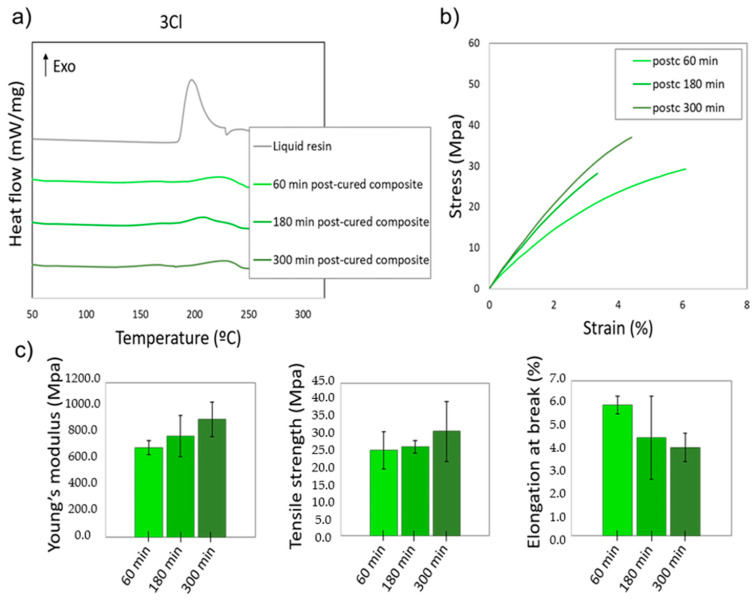
(**a**) DSC thermograms of the liquid resin and 3Cl composites subjected to UV post-curing processes at 60 °C and different times: 60, 180 and 300 min; (**b**) typical stress-strain curves and (**c**) average Young’s modulus, tensile strength, and elongation at break of the different post-cured nanocomposites.

In order to evaluate whether the resin with the larger amount of AgClO_4_ (3 wt%) could achieve a higher degree of cure to improve its mechanical properties, UV post-curing processes were carried out for longer times, in particular for 180 and 300 min. For these processes, the degree of cure was calculated by DSC, and its influence on the mechanical properties of the material was analysed. Figure 7a shows the DSC thermograms of the 3Cl nanocomposites after UV post-curing processes at 60 °C for 180 and 300 min. The curves corresponding to the liquid resin and to the post-curing process of 60 min are also included for clearer interpretation. As can be observed, the DSC peaks were reduced when the post-curing time increased. Hence, by increasing the post-curing to 300 min, an increase in the degree of cure up to 81% was calculated. This shows that for larger amounts of Ag precursor, in this case AgClO_4_, which are expected to consume a larger amount of photoinitiator obtaining a higher density of Ag NPs, the resin requires longer times to fully polymerize, and for curing times as long as 300 min (5 times larger than required for the unfilled resin) the photopolymerization process is still incomplete.

Figure 7b,c show the mechanical properties measured in the nanocomposites with UV post-curing processes of 60, 180, and 300 min. When the post-curing time was increased, the nanocomposites showed an improvement in the Young’s modulus of 20% (from 696.6 MPa to 914.9 MPa) and in the tensile strength of 10% (from 25.2 MPa to 30.7 MPa), with a slight reduction in elongation at break (5.9% to 4%). For SLA nanocomposites, lower crosslinking density induced lower mechanical properties and thus less rigidity and earlier failure. However, the exposition to UV light after the printing process continued the polymerization and, thus, the number of covalent bonds increased, enhancing the degree of cure and, consequently, the mechanical resistance of the material.

However, this improvement did not allow the values observed for the unfilled resin in Figure 5, or for the nanocomposites containing lower precursor concentrations, where the Young’s modulus and the tensile strength were approximately 15% and 37% larger, to be reached. As explained above, the likely reasons for this fact include both an incomplete curing of the resin and fragilization due to the presence of the Ag NPs. These results show that composites with higher concentration of Ag NPs, showing enhanced electrical/optical performance, hinder the successful photopolymerization of the acrylic resin, since there is an actual competition of the Ag precursor with the monomers for the photoinitiator. In this case, a compromise between the mechanical properties and the electrical/optical properties of the material should be acquired according to application specifications. Further strategies will be explored in order to obtain conductive resins without a detriment in their mechanical properties, such as including additional photoinitiator in the mixture prior to the SLA process.

## 4. Conclusions

In this paper, we demonstrated that the SLA process can simultaneously reduce Ag precursors (AgNO_3_ and AgClO_4_) to obtain in situ Ag NPs while polymerizing the liquid acrylic resin into a solid matrix of complex geometries. Our results show that the higher solubility of AgClO_4_ (compared to AgNO_3_) in the acrylic resin allows the formation of Ag NPs with size of few nms all through the material for the higher concentrations tested, which modifies the optical and electrical behaviour of the pristine resin. A remarkable reduction of four orders of magnitude in the electrical resistivity was achieved for the higher AgClO_4_ concentration studied (3 wt%). However, the competitive reaction for the photoinitiator did not allow the complete photopolimerization of the resin for larger precursor concentrations, which had a detrimental effect on the mechanical properties of the composites. Our results show that this could be partially compensated for by increasing the duration of the UV post-curing treatment, increasing both Young’s modulus and tensile strength. The results obtained in this work show that 3D printable acrylic based-nanocomposites containing Ag NPs are potential competitive alternatives in the electronic and optical markets due to the possibility of tailoring their physical properties. Knowledge on the relationship between the functional and the structural properties of these materials paves the way for further development in order to be used in such important fields today as biomedicine and aerospace.

## Data Availability

Not applicable.
